# Diagenetic processes in Quaternary fossil bones from tropical limestone caves

**DOI:** 10.1038/s41598-020-78482-0

**Published:** 2020-12-08

**Authors:** Daniel Vieira de Sousa, Estevan Eltink, Raquel Aline Pessoa Oliveira, Jorlandio Francisco Félix, Luciano de Moura Guimarães

**Affiliations:** 1 Colegiado de Geografia, Universidade Federal do Vale do São Francisco, Senhor do Bonfim, 48970-000 Brazil; 2 Colegiado de Ecologia, Universidade Federal do Vale do São Francisco, Senhor do Bonfim, 48970-000 Brazil; 3 Colegiado de Ciências dos Materiais, Universidade Federal do Vale do São Francisco, Juazeiro, 48902-300 Brazil; 4 Instituto de Física, Universidade de Brasília, Brasília, 70910-900 Brazil; 5 Departamento de Física, Universidade Federal de Viçosa, Viçosa, 36570-000 Brazil

**Keywords:** Palaeontology, Mineralogy, Characterization and analytical techniques, Microscopy

## Abstract

Quaternary fossils from limestone caves bear various diagenetic features due to the complex nature of sedimentary processes. However, few studies have addressed the problem of diagenetic changes in fossils from tropical-wet environments. We study Quaternary fossil bones from different sites of a tropical limestone cave in northeastern Brazil. These fossils show diverse diagenetic features. The approach encompassed the use of scanning electron microscopy, Raman spectroscopy, and X-ray diffraction to understand the modification of the fossil bone structure, chemical composition, and mineral assemblage during the diagenesis processes. We describe a model for fossil diagenesis in tropical limestone caves that involves early and advanced diagenetic stages, which produce two routes with different endmembers. The diagenesis in the cave alters the crystallinity and ordering of hydroxyapatite. The recrystallization of hydroxyapatite appears to be strongly influenced by dripping water that is rich in calcium carbonate, which leads to crystal formation with higher crystallinity. In the absence of calcium carbonate, hydroxyapatite diagenesis involves crystal growth but not necessarily dissolution of the original material, which enables remarkable preservation of the biological structure.

## Introduction

Fossil bones undergo modifications that are caused by endogenous and exogenous processes^1–5^. Knowledge of their chemical and mineralogical compositions may be a valuable source of information about the past.

Bone is a complex material that is composed of a biomineral phase, which is referred to as carbonate apatite (Ca_10_[(PO_4_)_6−x_(CO_3_)_x_](OH)_2_), and an organic matrix (mainly type I collagen)^6^, which is organized in a hierarchical architecture, from the nanoscale to the macroscale^7, 8^. In bones, the mineral structure usually changes in two distinct phases, i.e., first in vivo during an animal's life and after death. The timescale of the latter may be extended and includes the subject of taphonomy and fossil diagenesis^9^. During the taphonomic stage, microorganisms can attack the bones' organic and inorganic contents. The bone structure changes in the fossil diagenesis stage, which causes physical/chemical/mineralogical alterations at different scale levels^10–13^. A consequence of fossil bone changes during the diagenetic processes includes local mineral inclusions, which lead to absorbing of chemical elements or replacing the original biomineral composition with secondary minerals. The results are destructive to the original bone histological structure^4, 14^.

Despite numerous fossil diagenesis investigations, few physico-chemical models concern bone structure and composition. Most of these models are focused on temperate and tropical arid environments^1, 15, 16^. Furthermore, fossil diagenetic studies focus on archaeological human bones or old paleontological remains from periods such as the Cretaceous and Eocene^3, 4, 17–22^. Few studies have addressed the diagenetic processes in tropical-wet environments^23^ and disregard hydroxyapatite lattice alterations. The diagenetic process in fossil records is site-specific and dependent on geochemical conditions^1, 10, 23, 24^. The study of diverse site environments fills a gap in the knowledge about the wide variety of diagenetic processes. Here, we focus on fossil diagenesis in tropical limestone caves.

Diagenetic parameters that are used to study fossil diagenesis include (1) collagen content, (2) histological integrity, (3) porosity, and (4) crystallinity. To understand the fossilization process and assess the molecular composition and modification, optical microscopy (OM), scanning electron microscopy and transmission electron microscopy (SEM and TEM, respectively) associated with microprobe analyses^5, 21^, and  fourier transformation infrared spectroscopy (FT-IR) and raman spectroscopy are powerful tools^3, 5, 6, 25–27^. Many researchers have also applied X-ray diffraction (XRD) and synchrotron-based techniques to study the mineral bone matrix^13, 28–32^.

Quaternary fossils that are recorded in limestone caves have distinct diagenetic features due to the complex nature of the sedimentary processes^33–37^. In these fossils, the presence or absence of mineral incrustation, such as calcium carbonate minerals, iron, and manganese oxides, has a key role in diagenesis. Diagenesis is caused by a combination of water composition, biological activity, and site environmental conditions^1, 5^. These different processes lead to biofilm formation, precipitation of minerals, and substitution of the organic mineral matrix with secondary minerals within the bone structure^38^. However, the details of the physical–chemical process in Quaternary fossil bone found in limestone caves still demand more clarification.

**Figure 1:**
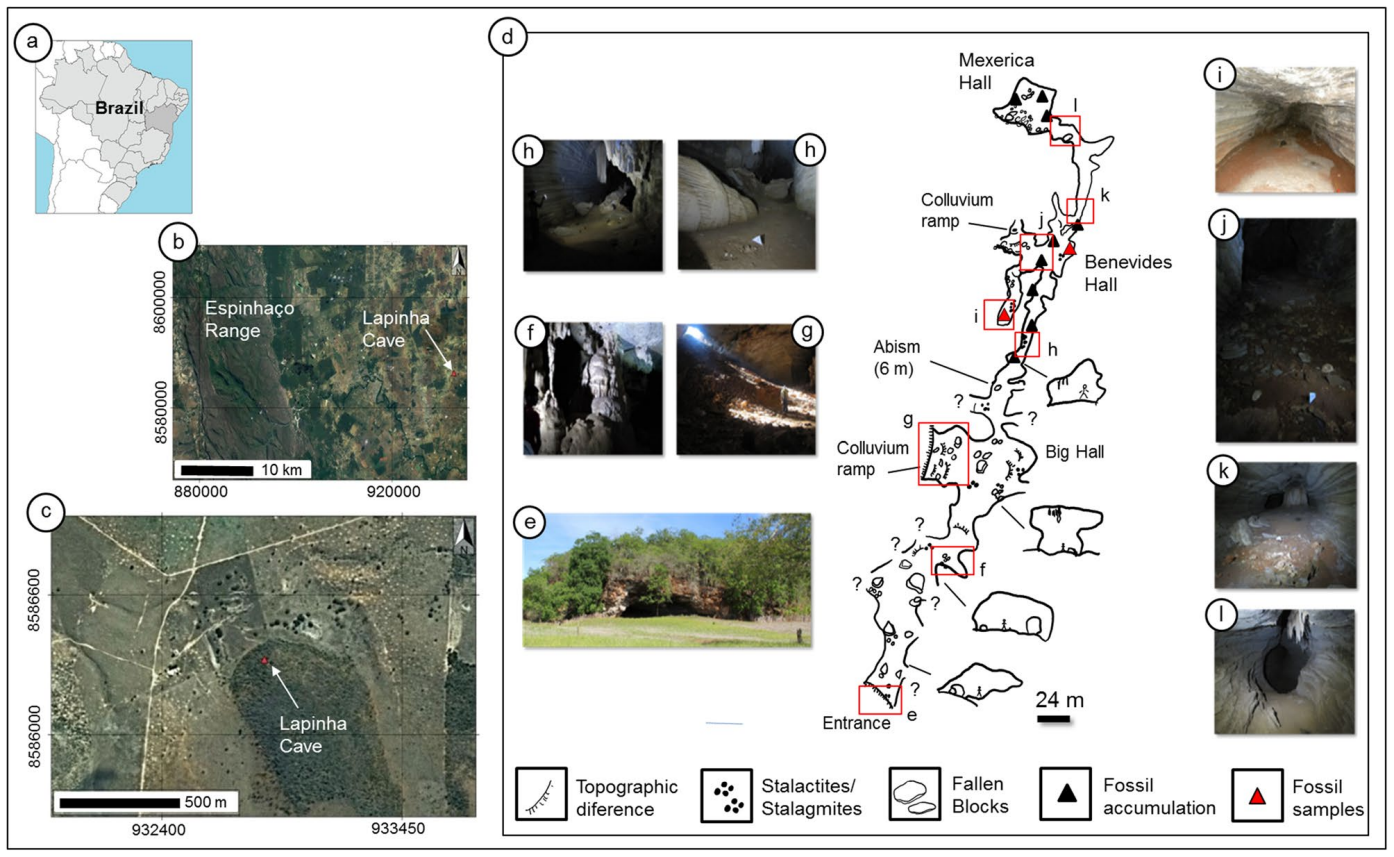
Detailed (**a**), (**b**), and (**c**). Location of Lapinha cave. Figure (**d**) shows a map of the cave, in which the entrance and different locations are shown with their corresponding photos (**e**–**l**). The red triangles show samples that were collected at the fossil-bearing sites.

This study investigates Quaternary fossil bones that were collected from the limestone cave named ‘Lapinha’ in Nova Redenção, Bahia, Brazil (Fig. 1). The fossil bones and cave were discovered by the Environmental Group of Protection, Paleontology and Speleology (GAPPE) from Nova Redenção. This cave is located in the Una-Utinga Basin and formed by sedimentary rocks from the Neoproterozoic (São Francisco Supergroup, Una Group) in the geotectonic domain of Chapada Diamantina^39^. The carbonate facies of the Una-Utinga Basin forms a broad karstic system with copious limestone caves. Cartelle et al.^40^ and other researchers^41–43^ described Quaternary fossils from these caves, including a diversity of giant sloths and other mammals. In the Lapinha cave, the fossil record includes postcranium, tooth, mandibles, and fragmentary skulls of mammals, such as the ground sloth genera (*Catonix*, *Valgipes,* and *Nothrotherium*), cervid (*Mazama*), felid (*Smilodon*), and tapirid (*Tapirus*)^44^.

To investigate the fossils’ diagenetic processes, we chose to cover the fossil diagenetic features that are commonly found in limestone Brazilian caves^42–45^. We employed a methodological strategy similar to that adopted by Reiche et al.^16^ to study the Neolithic site of Bercy in Paris, France. We selected two representative samples from the fossil assemblage from the Lapinha cave. The first sample comprised a ground sloth vertebra (*LAPA/SBF-3-0017*) that was collected in the lateral conduit alongside the colluvium ramp with stalactites (Fig. 1d,i). *LAPA/SBF-3-0017* consists of isolated bone that is half-covered by sediments and incrusted by a thick layer of calcite. The second sample also includes a ground sloth vertebra (*LAPA/SBF-3-0081*) that was discovered within the disarticulated skeleton of *Valgipes bucklandi*. We collected this sample in the 'Benevides Hall' (Fig. 1d,j), which is partly absent of stalactite/stalagmites.

The sample's postmortem histories produce similarities in the taphonomic features, such as weathering stages, abrasion levels, absence of lichen impression, and breakage types (Supplementary Table S1). However, the samples passed by other diagenetic histories, which guide different geochemical and recrystallization pathways^1^.

This research aims to employ several methods, such as optical microscopy, scanning electron microscopy, Raman spectroscopy, and X-ray diffraction, to understand the modification of the fossil bone structure, chemical composition, and mineral assemblage during the diagenetic processes of Quaternary fossils in a tropical limestone cave. Our data show the correlation of apatite lattice defects with diverse diagenetic pathways, which leads to recrystallization or nucleation. We propose a physico-chemical model of fossil diagenetic processes in limestone caves with tropical-wet environments from our data.

## Results

### Raman spectroscopy analysis

**Figure 2:**
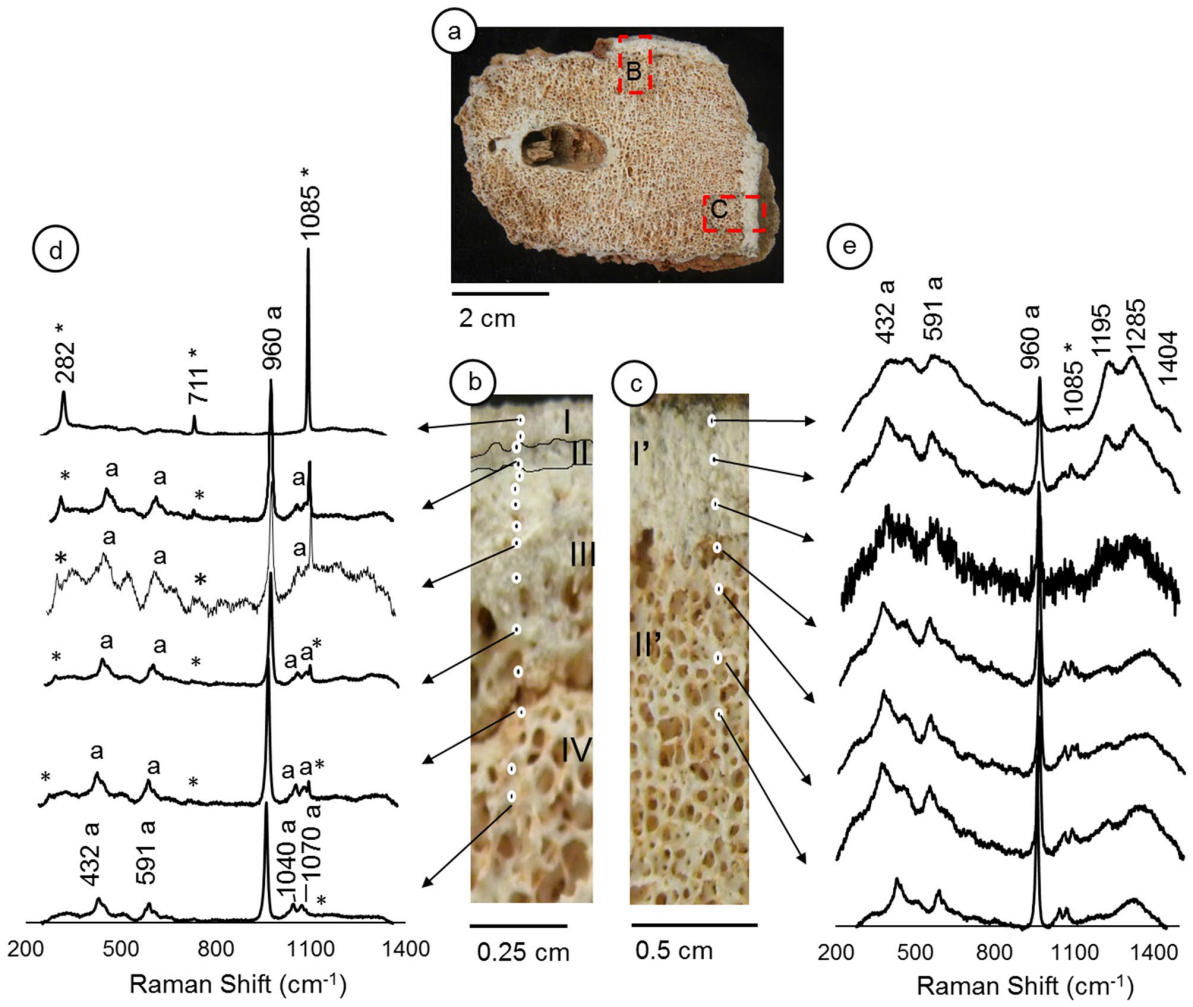
Internal surface of the LAPA/SBF-3-0017 sample. Figure (**a**)—shows the selected areas (red dashed boxes B and C) where micro-Raman measurements were carried out. Figures (**b**) and (**c**)—Details of red dashed boxes in figure a. Figures (**d**) and (**e**)—Raman spectra of each microstratigraphic ‘layer’; ‘*’ indicates the peaks for calcite, and 'a' refers to hydroxyapatite.

Raman spectroscopy analysis of the *LAPA/SBF-3-0017* sample’s external surface revealed five features that were formed by different mineral incrustations (Supplementary Fig. S2). The first feature, which corresponds to the outer covering of the fossil, is formed by calcite precipitate and identified by band assignments of v_1_ (1085 cm^−1^, symmetric stretching), v_2_ (712 cm^−1^, lattice modes), and v_4_ (780 cm^−1^ in-plane bendings). The coexistence of the bone mineral matrix (hydroxyapatite 960 cm^−1^, P-O symmetric stretching) and carbonate mineral (calcite) in Layer I indicates the formation of a thin calcite layer, which suggests that dripping water was not intense (Supplementary Fig. S2). Feature “II” represents a fine sand material that adheres to the outer surface of the fossil bone, which is exclusively composed of well-crystallized calcite (shown by vibrational modes of v_1_, v_2,_ and v_3_). Feature "III" is similar to feature "I"; however, it has higher calcite precipitation on the bone, which causes a low intensity of phosphate peaks. Feature IV is a thin accumulation of fine calcite sand; however, it bears a very notable v_1_ peak of apatite (960 cm^−1^). The spectrum of feature V (Supplementary Fig. S2) reveals mineral fractions of haematite (410 and 1320 cm^−1^) and calcite (1085 cm^−1^). Regarding the *LAPA/SBF-3-0017* sample’s internal surface, the first observation is the presence of a reddish colour in the bone pores, which shows iron oxide impregnation. The bone shows two regions with different morphologies, as analyzed by micro-Raman spectroscopy-denominated regions B and C (Fig. 2a–c).

Region B of the *LAPA/SBF-3-0017* specimen shows four internal sequences, which are denominated as ‘layers’ I, II, III, and IV (Fig. 2b). In ‘layer I,’ the vibrational modes of v_1_, v_2_, and v_3_ of calcite minerals are observed, which reinforces that only calcite was precipitated and formed a crust on the fossil surface that originates from the drip of water (Fig. 2d). In layer II, the band assignments of v_1,_ v_2_, and v_4_ (430, 590, and 960 cm^−1^, respectively) indicate the presence of calcite and biogenic apatite. ‘Layers III and IV’ represent the cortical and cancelous bone matrix, which shows a similar composition to ‘layer II’; ‘layer III’ has a considerable infilling of calcite in the pores; and ‘layer IV’ has calcite impregnation, which forms a carbonate coating on the bone matrix. In layer IV, we can observe this coating with a hypocoating of clay, silt particles composed of Fe oxides and quartz grains (Supplementary Fig. S1). However, we note the lack of a vibrational mode for iron oxide minerals.

In the analyses of region C (Fig. 2c), the intensity of fluorescence increases closer to the outer surface. The peaks related to calcite are very discrete and different from the vibrational modes of phosphate (Fig. 2e). There are unidentified peaks at frequencies of 1085, 1195, 1285, and 1404 cm^−1^ in the sample (Fig. 2e). The peaks are more intense and were not observed in region 1 (Fig. 2d).

**Figure 3:**
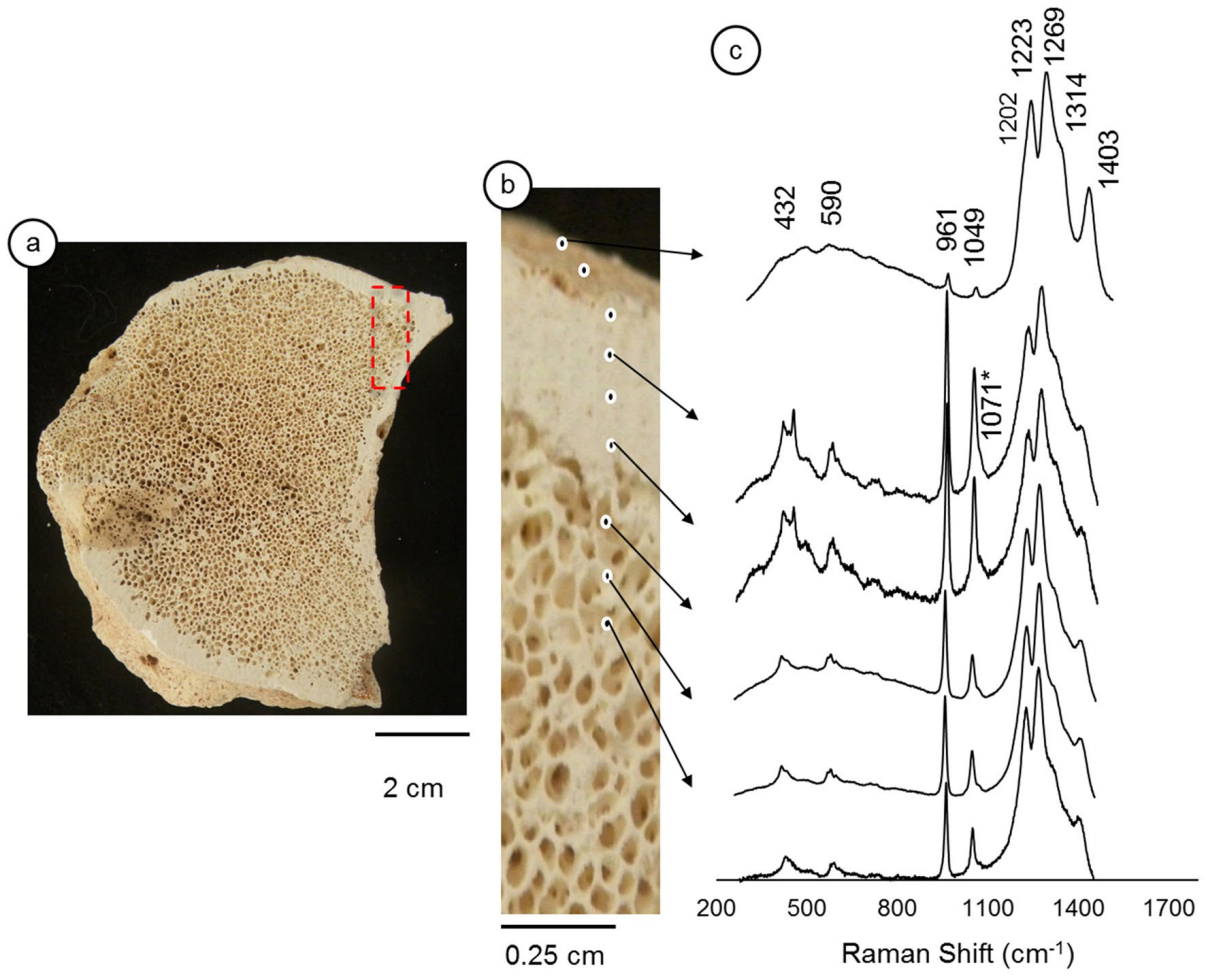
Internal surface of the LAPA/SBF-3-0081 sample. Figure (**a**)—Red dashed box indicates the analyzed area in the sample. Figure (**b**)—Details of the analyzed area and the micro-Raman measurement points. Figure (**c**)—Raman spectra, where ‘*’ indicates the B-type carbonate group.

The Raman spectroscopy of the *LAPA/SBF-3-0081* sample (Fig. 3a,b) reveals a homogeneous mineralogical composition of hydroxyapatite v_1_ (961 cm^1^), v_2_ (432 cm^−1^), v_3_ (1049 cm^−1^), and v_4_ (590 cm^−1^) and a lack of diagenetic features, as shown in the *LAPA/SBF-3-0017* sample (Fig. 3c). Regarding the Raman spectra, the *LAPA/SBF-3-0081* sample's remarkable feature is the greater intensity of v_3_ PO_4_^−3^ (1049 cm^−1^). We did not observe calcium carbonate minerals of diagenetic origin (v_1_-CO_3_^2−^ 1085 cm^−1^). This absence confirms that this sample was not affected by drip water that is rich in calcium carbonate. Similar to the previous sample, unidentified peaks appear in the high-energy region. These peaks were also reported by Piga et al.^46^, who did not mention their origin.

### Scanning electron microscopy (SEM) and geochemical mapping by energy dispersive X-ray spectroscopy (EDS)

**Figure 4:**
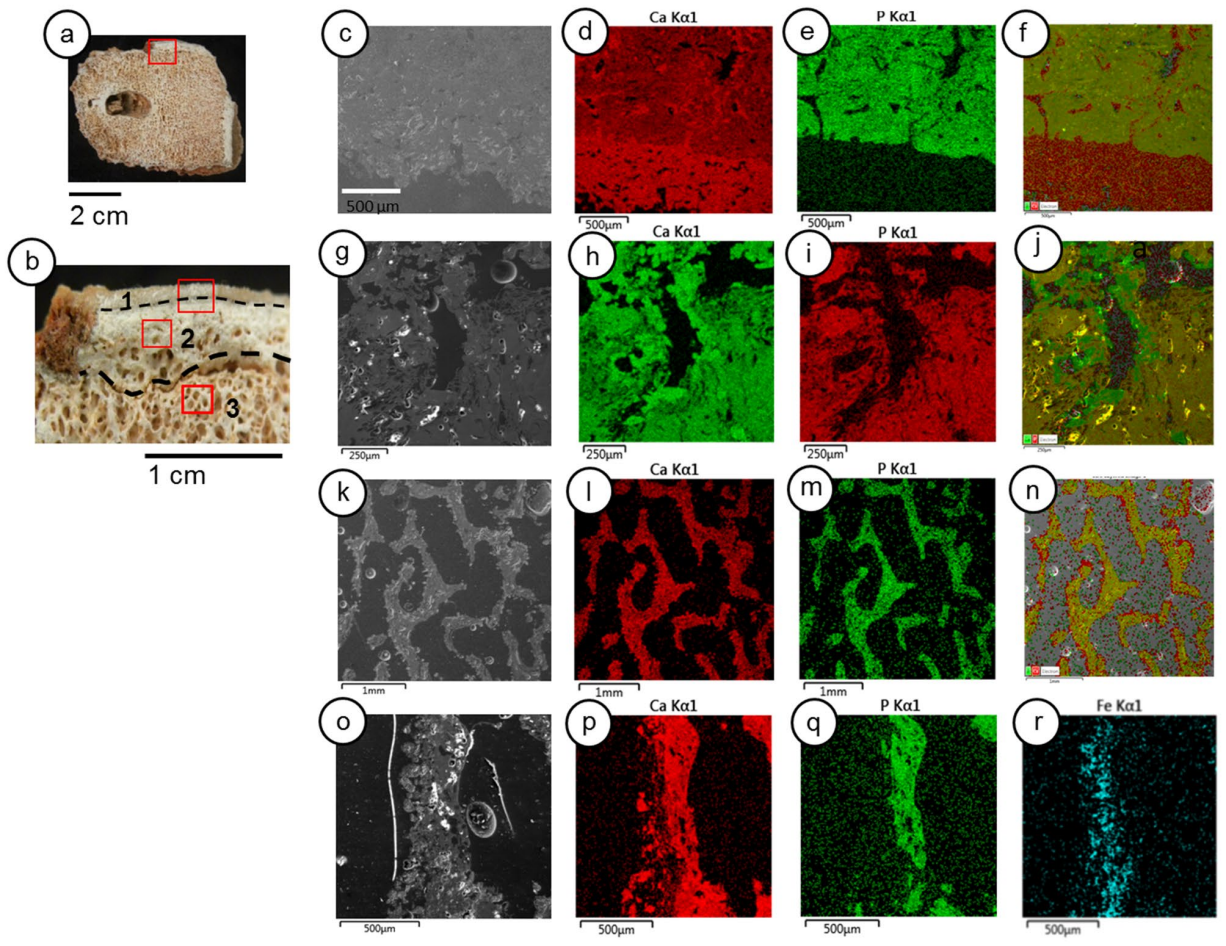
Scanning electron microscopy (SEM) and geochemical mapping by energy dispersive X-ray spectroscopy (EDS) of the LAPA/SBF-3-0017 sample. (**a**)—Internal surface of the sample with the selected area for analyses (red box). (**b**)—Red box in detail shows the three different selected areas (red boxes) for SEM analysis: layer 1—external surface of the bone (**c**–**f**); layer 2—cortical and intermediate layers (**g**–**j**), layer 3—internal cancellous tissue (**k**–**n**), in more detail (**o**–**r**). (**a**)—SEM image of calcite precipitate layer 1 and part of the cortical bone, layer 2. (**d**, **e**) Ca and P distributions. (**f**)—Both Ca and P in the same mapping; note the intense Ca infillings inside the cortical bone pores. (**g**)—Backscattered electron image of the cortical bone, layer 2. (**h**, **i**)—Ca and P distributions. (**j**)—Ca and P in the same mapping, observe the calcite precipitate in the pores. (**k**)—SEM image of the cancellous bone, layer 3. (**i**), (**m**)—Mapping of Ca and P, respectively. (**n**)—Both Ca and P in the same mapping. (**o**)—Detailed SEM image of the cancellous bone surface. (**p**, **q**)—Geochemical mapping of Ca and P, respectively. (**r**)—Mapping of iron.

SEM–EDS shows details of calcite precipitates in the *LAPA/SBF-3-0017* sample (Fig. 4). The bone's external surface underwent considerable calcite precipitation (Fig. 4a–f). Inside the cortical bone layers, there are calcite dense and continuous infillings (Fig. 4g–j); however, they are lesser than the edge of the cortical bone (Fig. 4d–f). The intensity of carbonate precipitation in the cancellous bone structures decreased significantly (Fig. 4k–n). However, the inner parts of the bone demonstrate a substantial presence of iron, which shows that aloctonous terrigenous composition, such as clay-coating, interacts in the cancellous bone (Fig. 4o–r).

**Figure 5:**
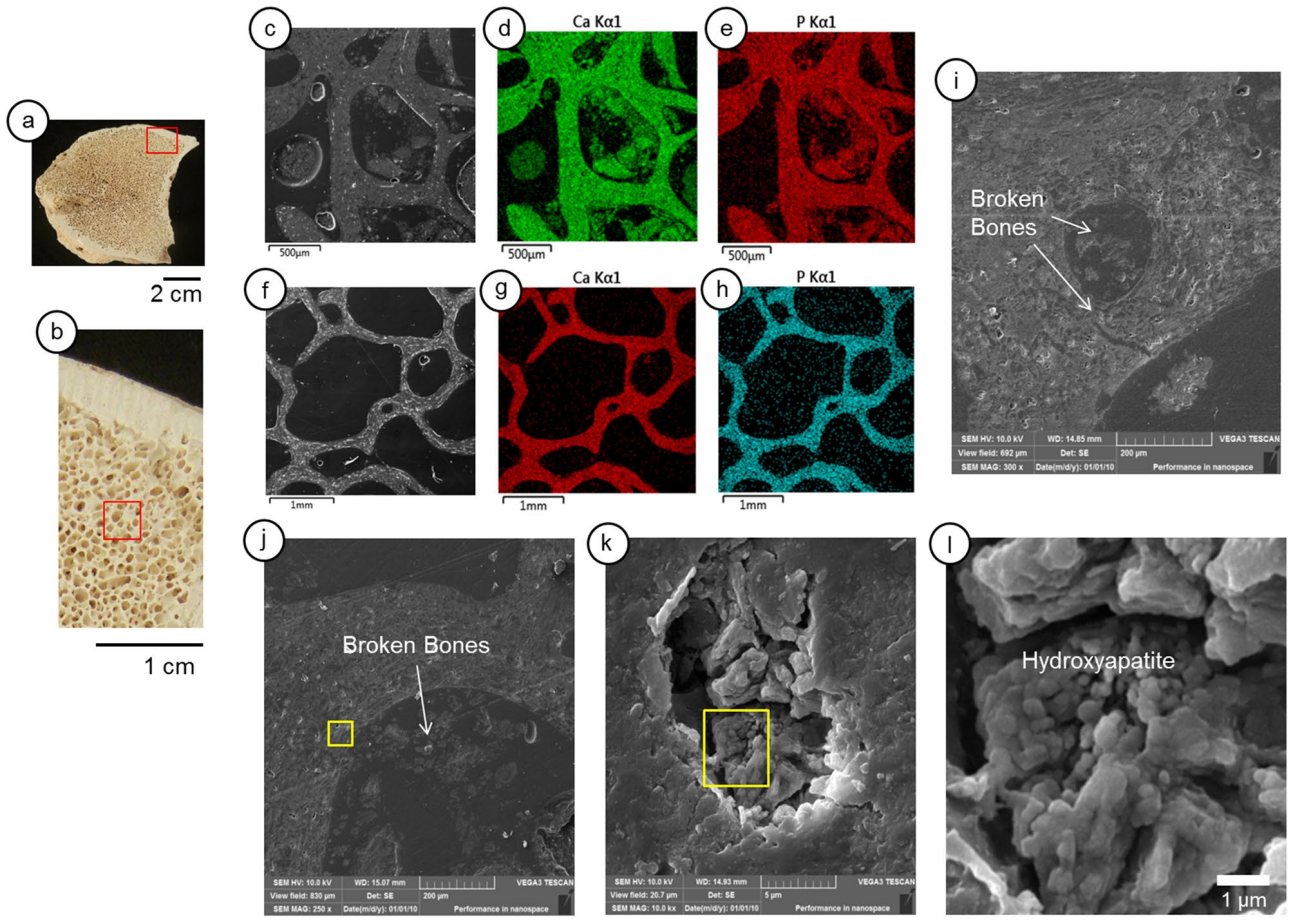
Scanning electron microscopy (SEM) and geochemical mapping by energy dispersive X-ray spectroscopy (EDS) of the LAPA/SBF-3-0081 sample. (**a**)—An overview of the internal section of the sample, showing the analyzed area (red box). (**b**)—Detail of the analyzed area, which indicates the specific region for SEM and EDS analyses (red box). (**c**, **d**)—Backscattering images of SEM where microchemical mappings were constructed. (**e**, **f**)—Calcium micro-mappings. (**g**, **h**)—Phosphorus micro-mappings. (**i**)—Microstructure in which bone fragments and breakages appear near osteocytes. (**j**)—SEM image details the bone structure and broken bone fragments inside the porous system. The yellow box details the bone structures. (**k**)—Detailed SEM image of the Haversian canals its hydroxyapatite structures. (**l**)—SEM image of the hydroxyapatite structures.

The *LAPA/SBF-3-0081* sample (Fig. 5a,b) mainly differs in the exogenous calcium deposition (Fig. 5c–h). The bone shows an absence of fossil diagenetic signals, which resemble an intact bone (Fig. 5f). However, the *LAPA/SBF-3-0081* sample shows a significant presence of cracks and broken bone fragments inside the porous system (Fig. 5i–l). This feature may be due to the transport process into the cave or even during bone necrolysis. In Fig. 5k,l, it is possible to observe hydroxyapatite microstructures inside the Haversian channels.

### X-ray-diffraction analyses

**Figure 6:**
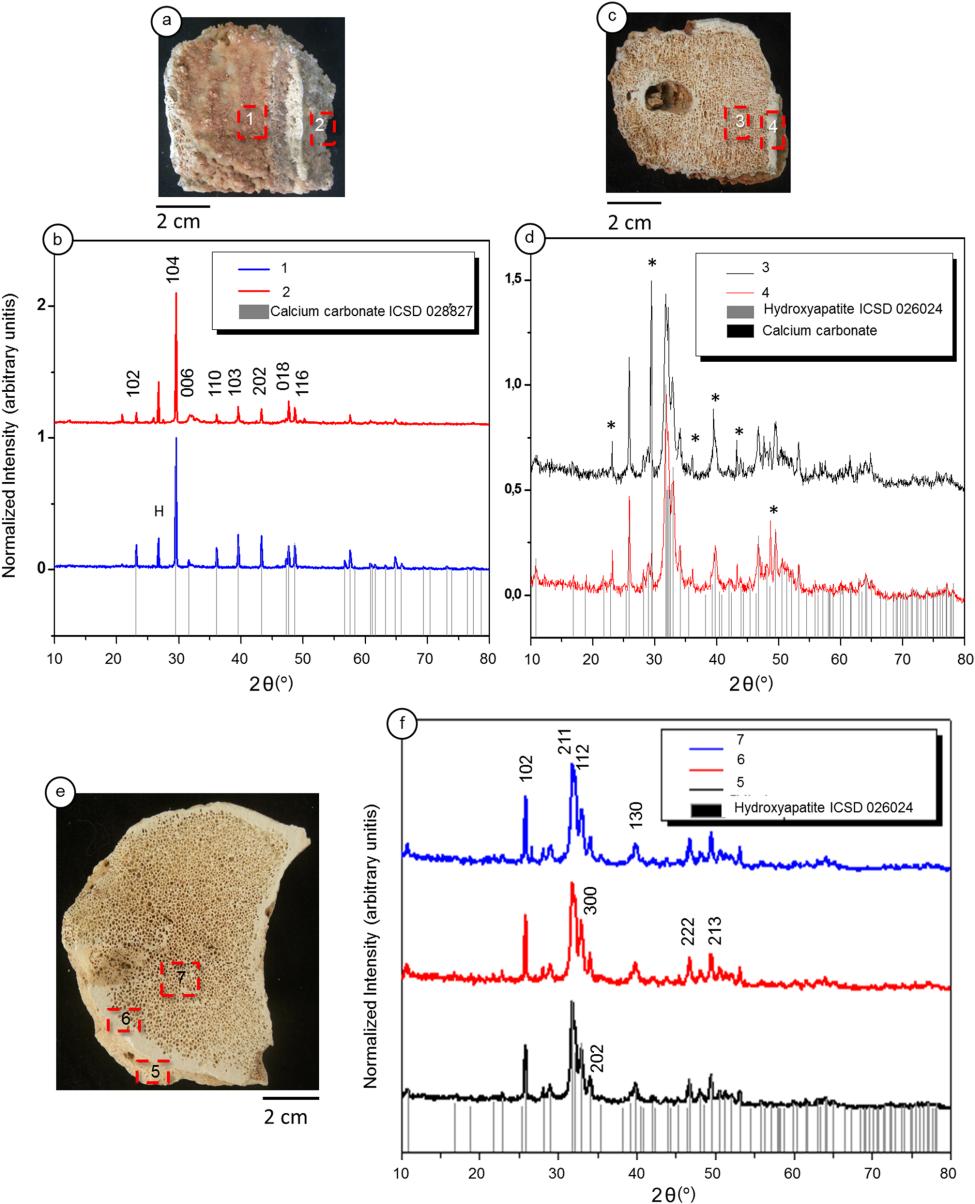
X-ray diffraction (XRD). (**a**)—Image of the external surface of the LAPA/SBF-3-0017 sample. The red dashed boxes (areas 1 and 2) indicate the bone location where the data were collected, as represented in the diffractogram. (**b**)—Diffractograms of the sample external face. (**c**)—Image of the internal surface of the sample. The red dashed boxes show the locations of the data represented in the diffractogram, in which area 3 focuses on the cancellous bone and area 4 focuses on the cortical bone. (**d**)—Diffractograms of the internal surface of the sample. ‘*’ indicates peaks for calcite. Reference standards for ICSD 026204 hydroxyapatite and calcium carbonate ICSD 028827. (**e**)—Image of the LAPA/SBF-3-0081 sample. The red dashed boxes represent the location of the data represented in the diffractogram, in which area 5 focuses on the external surface, area 6 focuses on the cortical bone, and area 7 focuses on the cancellous bone. (**f**)—Diffractograms of the internal and external surfaces of the LAPA/SBF-3-0081 sample. The numbers in the diffractograms represent the hkl plane. The diffractogram images were constructed by X-Pert HighScore software (Panalytical).

We analyzed the samples by X-ray-diffraction XRD in distinct regions. For the *LAPA/SBF-3-0017* sample, we conducted four measurements at different sites. Sites 1 and 2 are located in diverse regions of the external face, and sites 3 and 4 are located in the internal face of cancellous bone and cortical bone, respectively. The experimental diffractograms of sites 1 and 2 (Fig. 6a) match calcite with a rhombohedral crystal structure and space group R-3c (ICSD 028827) (Fig. 6b). The crystallographic parameters of calcite are listed as follows: a = 4.9803 Å, b = 4.9803 Å, c = 17.0187 Å, α = 90°, β = 90°, γ = 120°, density 2.73 g/cm^3^, and volume 365.057 × 10^6^ pm^3^. In terms of the crystallite size, sites 1 and 2 (external surface of *LAPA/SBF-3-0017*) show a minute variation. Using the Scherrer equation (Supplementary Table S2), we discovered that site 1 has calcite with a domain of crystallite size within 51.03 nm, and site 2 has calcite with a domain of crystallite size within 50.73 nm (Fig. 6a,b).

The XRD results of the *LAPA/SBF-3-0017* sample's inner face have identical compositions and crystal structures (Fig. 6c,d). The data show a hydroxyapatite composition (Ca_10_ (PO_4_)_6_ (OH)_2_) with a hexagonal crystal structure and space group P63/m. The crystallographic parameters are a = 9.4240 Å, b = 9.4240 Å, c = 6.8790 Å, α = 90°, β = 90°, γ = 120°, density 3.15 g/cm^3^, and volume 529.09 × 10^6^ pm^3^ (reference sheet ICSD 026204). The hydroxyapatite crystallite size is larger than that reported in the literature for unaltered bones^6, 47^. The data obtained from the cortical bone (Fig. 6c, site 4) demonstrate a crystallite of 39.26 nm, and the cancellous bone region is 41.08 nm (Fig. 6c site 3). Perhaps these values are not significantly different, but according to Handschin and Stern^48^, the variation in crystallinity correlates to tissue type. In the *LAPA/SBF-3-0081* sample, the unique mineral phase was hydroxyapatite like *LAPA/SBF-3-0017* sample (reference sheet ICSD 026204), although they exhibit a significant difference in terms of crystallite size (Supplementary Table S2). The cortical bone region shows a crystallite size of 30.93 nm, while the size of the cancellous bone is 31.15 nm. The measurements taken of the *LAPA/SBF-3-0081* sample’s external surface have a larger crystallite with a size of 33.33 nm (Fig. 6e,f).

## Discussion

### Diagenetic processes and routes

**Figure 7:**
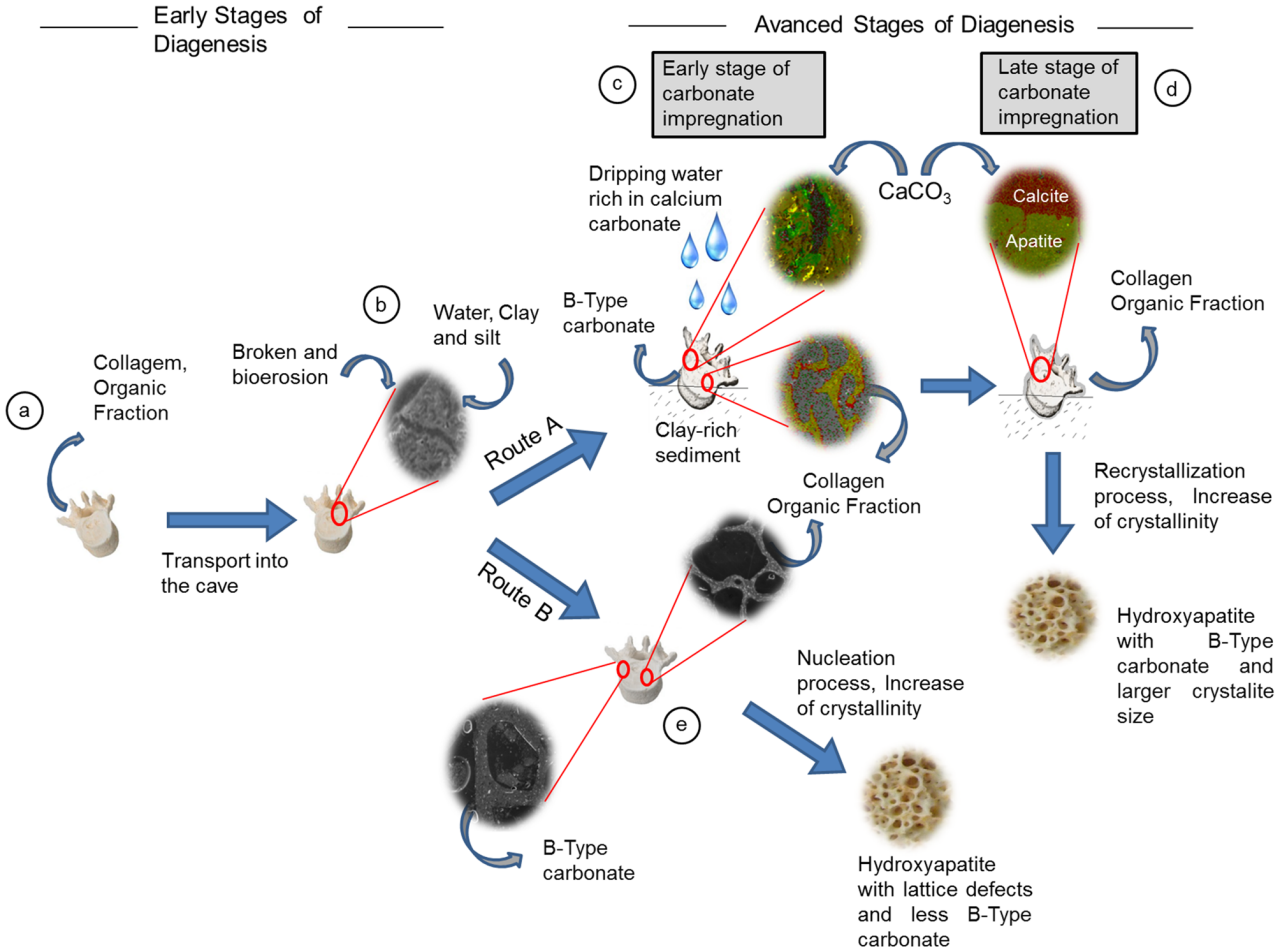
Model for diagenesis routes in Quaternary fossils from tropical limestone caves. (**a**)—The initial phase is related to the time soon after an animal's death, in which organic fraction degradation occurs. (**b**)—The early stage of diagenesis is followed by processes of transport and deposition inside the cave. (**c**)—Advanced stage of diagenesis (route A), in which the initial entry of dripping water that is rich in calcium carbonate and precipitation of calcite occurs on the bone. (**d**)—Advanced stage of diagenesis, in which the well-marked process of calcium carbonate mineral precipitation (permineralization) occurs with intense infilling of precipitated minerals in the pores of bone. Recrystallization of hydroxyapatite also occurs. (**e**)—Advanced stage of diagenesis (route B), in which continuous degradation of the organic fraction occurs, and hydroxyapatite crystal recrystallization occurs by the nucleation process. Due to the lack of dripping water that is rich in calcium carbonate, some of the type B carbonate binding sites of the formed hydroxyapatite crystals are not filled.

These results enable us to propose a diagenetic model for Quaternary fossils in tropical limestone caves (Fig. 7). Earlier studies show some aspects of our model. Weiner et al.^49^ demonstrate the effect of dripping water on the patchy preservation of bones at Hayonim Cave (Israel). Karkanas^50^ and Weiner^51^ show the relation of bone diagenesis and crystallite sizes, and some pathways described here are similar to Dal Sasso et al.^52^ for Mesolithic and Neolithic burial sites in Al Khiday, Sudan.

The first postmortem changes in the bone occur in the early stage of diagenesis (Fig. 7a), which comprises organic bone matrix degradation by microorganisms^15, 53, 54^. This decomposition will occur before and during transport and sedimentary events.

In the early stage of diagenesis, wet or dry conditions inside the cave drive bone organic phase degradation. In wet conditions, fungi, bacteria, and other microorganisms attack the organic phases, promote bioerosion features, as pointed out by Trueman et al.^55^, Fernández-Jalvo et al.^14^, Dal Sasso et al.^52^, Mayer et al.^23^, and others^5, 56–58^. Moreover, microorganisms produce organic acids as endmembers of the decomposition process^57, 59^; this acidification promotes the dissolution of hydroxyapatite and alters its biogenic structures^38^. In dry or low wet conditions, microorganism action is less marked, which produces more conserved histological structures. In this case, the hydrolysis process will decompose the organic fractions^59, 60^.

Post-depositional processes in the cave environment depend upon various site conditions, such as sediment mineralogical composition, pH, temperature, and water characteristics^1, 38^. Structural and organic fraction degradation generally takes a few years, and crystallographic alteration of the (bio)mineral fraction may occur during this process^13, 61^.

The LAPA/SBF-3-0017 sample (Fig. 3b) had a significantly altered histological microstructure and a small collagen quantity in the SEM analyses. We observed different conditions in the *LAPA/SBF-3-0081* sample, which preserves histological features, with some cracks and broken bones inside the pores (Fig. 4a,d,e,h).

**Figure 8:**
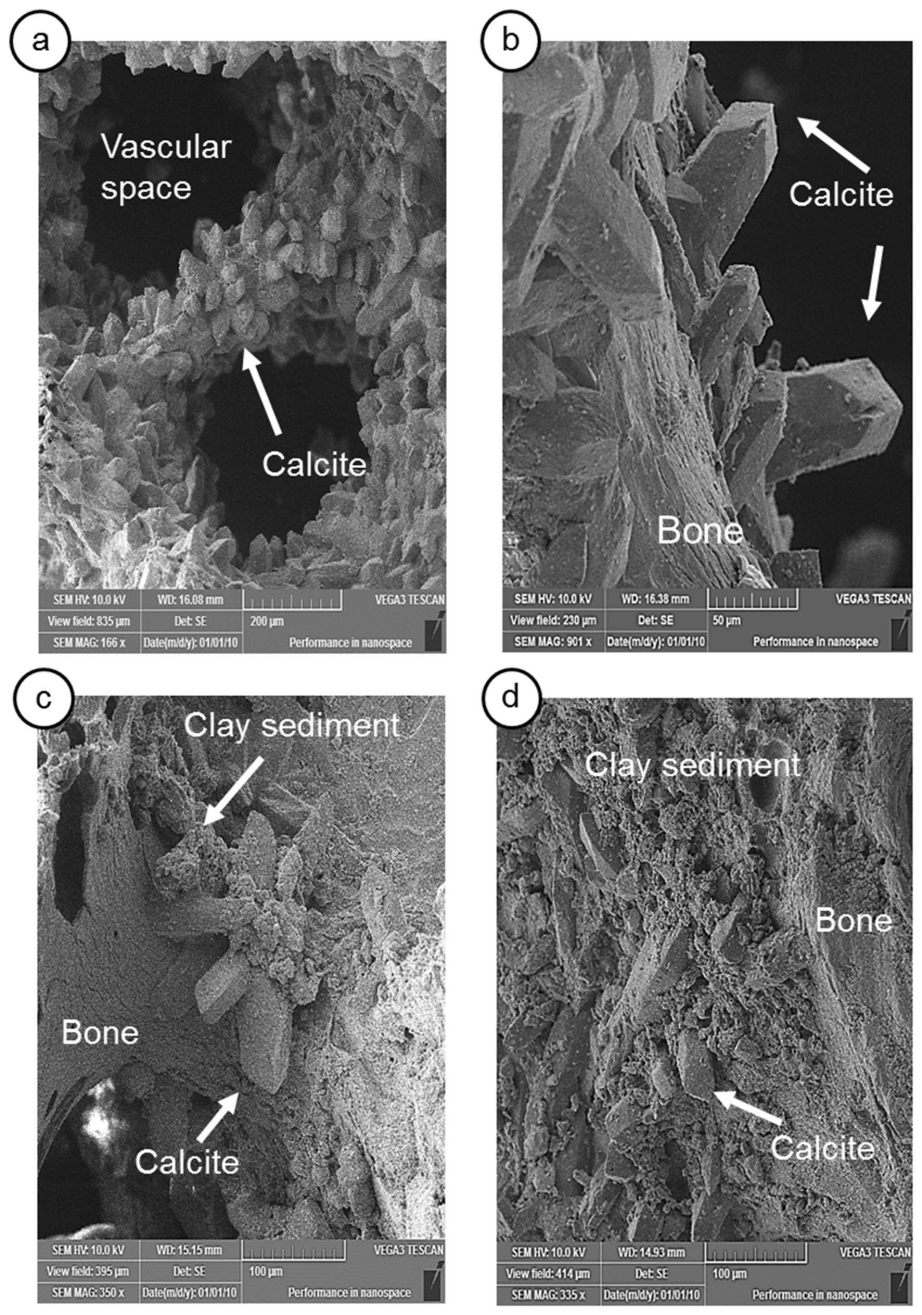
Scanning electron microscopy images of the LAPA/SBF-3-0017 sample. (**a**)—Vascular structure, in which it is possible to observe the intense precipitation of calcite crystals. (**b**)—Well-developed calcite crystals on the surface of the bone structure. (**c**, **d**)—Bone surface with precipitated calcite crystals that are partially covered by terrigenous material.

After distinct taphonomic pathways throughout the early stage of Diagenesis, a split into distinct routes—referred to here as route A and route B—occurs (Fig. 7b,c), which creates different advanced stages of diagenesis with distinct endmembers of taphonomic features. In addition to the decomposition of the organic matrix and alteration of the histological structure, the characteristics of the advanced stage of diagenesis of route A are the precipitation of well-developed calcite crystals inside the bone porous space, as noted by Fernández-Jalvo et al.^12^ (Fig. 8a–c, and Supplementary Fig. S4), resulting from dripping water that is rich in calcium carbonate (Fig. 7c). As a sign of successive input of terrigenous sediments, there is an extensive coating of iron oxides on this well-crystallized calcite in the porous space (Fig. 8c,d, and Supplementary Fig. S4). The granular microstructure of the sedimentary material in the bone porous space (Supplementary Fig. S1) suggests the erosion of well-developed soil mantles, such as Oxisols and Acrisols, with kaolinitic and oxidic mineralogy (Supplementary Fig. S5 and Supplementary Table S3), which indicates that the external environment of the cave had extreme weathering conditions.

In addition to calcite precipitation in the bone, hydroxyapatite's structural modification characterizes the advanced diagenesis stage. The diagenetic processes cause crystallographic changes in the mineral matrix^2, 13, 18, 62, 63^. According to Karkanas^50^, Weiner^51^ and Dal Sasso et al.^5^, the diagenesis process will form hydroxyapatite crystals with larger crystallite sizes and fewer carbonate groups^5, 64^. Hydroxyapatite minerals have two known carbonate binding sites that are referred to as A-type and B-type carbonate; A-type carbonate occupies a position at the edge of the crystal structure, while B-type carbonate has an inside position of the structure^6, 65^.

Diagenesis promotes the loss of B-type carbonate^13, 38^. For this reason, the rates of PO_4_^3−^ and B-Type CO_3_^2−^ are used to evaluate the diagenesis process in fossil bones^6, 18, 66–68^. In Raman scattering, only the B-type carbonate band is present^26, 69^ and appears at 1070 cm^−1^.

**Figure 9:**
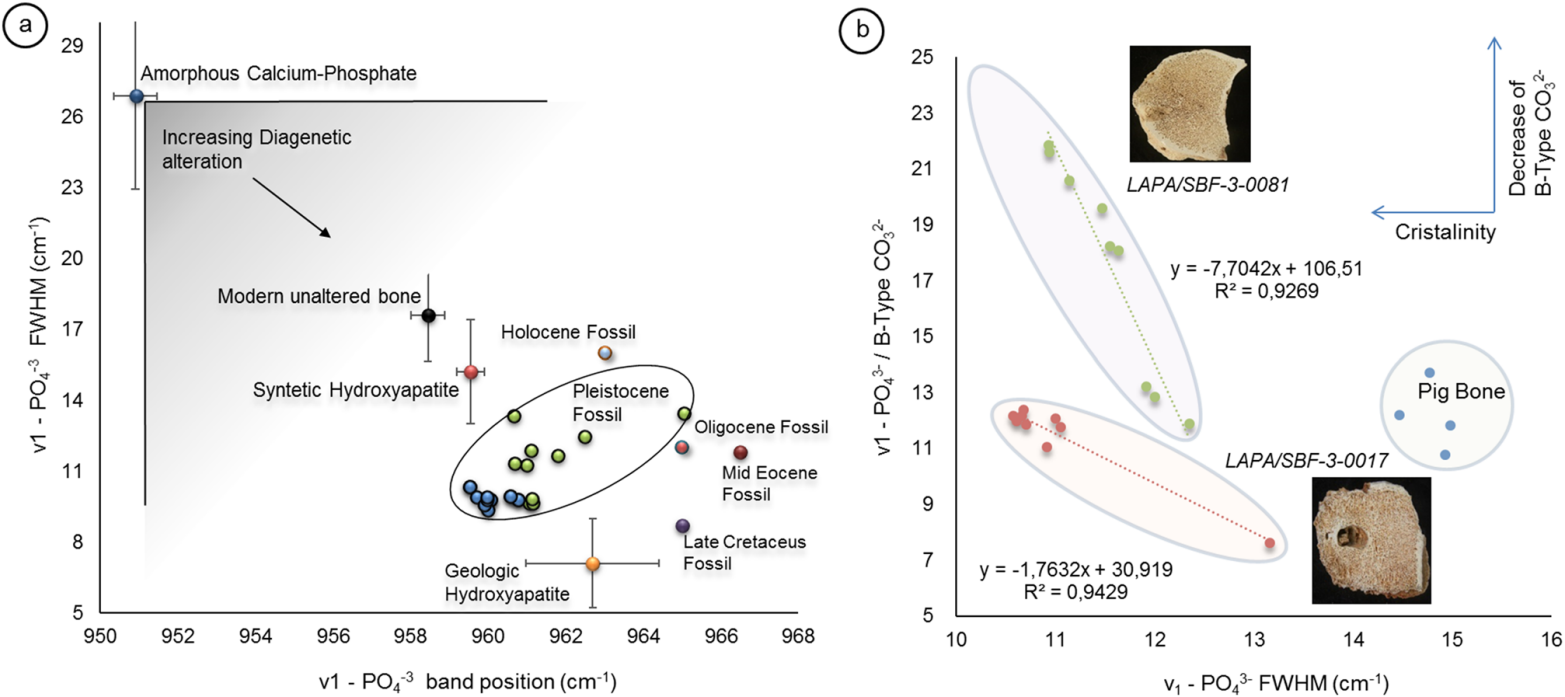
Characteristics of the Raman spectra of the *LAPA/SBF-3-0017* and *LAPA/SBF-3-0081* samples. (**a**)—Band position of v1-PO_4_^3−^ about its peak width at half-height of the data obtained in this research (Pleistocene fossils, points green and blue in image ‘**a**’) compared with available data in the literature (Supplementary Table S4). (**B**)—Relationship between crystallinity (FWHM of v1-PO_4_^3−^) and the existence of B-type carbonate within the bone structure (v1-PO_4_^3−^/B-type CO_3_^2−^).

To understand the effect of diagenesis concerning the Raman spectra of hydroxyapatite, we performed an analysis of our data with available data gathered from the literature (Fig. 9a and Supplementary Table S4). From the Raman spectrum of fossil bone, it is possible to observe that diagenesis translates to a decrease in the FWHM, the position change of the primary phosphate band, v_1_ peak of PO_4_^3−^, which represents the c-axis length of the unit cell^3, 26, 70^ (Fig. 9a), and the relative intensities of the B**-**type CO_3_^2−^ (Fig. 9b). The symmetric stretching of PO_4_^−3^ is sensitive to ionic impurities, and any ionic constituent change can affect the peak characteristcs^3^. FWHM values are inversely proportional to crystallite size^71^, so a narrower FWHM corresponds to higher crystallinity^29, 31, 70, 72^. The change in position of the v_1_ peak of PO_4_^3−^ (blueshift or redshift) provides strong evidence for ionic substitutions into hydroxyapatite and alters the crystal field lattice-bound phosphate, which contributes to an increasing atomic disorder^73^ (Fig. 6). Low values of the v_1_**-**PO_4_^3−^/B**-**type CO_3_^2−^ ratio would indicate either secondary hydroxyapatite precipitation or structural carbonate group loss.

Conversely, the increased values suggest either the substitution of secondary carbonate for biogenic phosphate or the dissolution of hydroxyapatite^73^. For ideal hydroxyapatite recrystallization, the availability of calcium and phosphorus is necessary. However, low calcium conditions imply that other ions can produce chemical bonds, such as Na, F, and Sr. Newly formed apatite crystal-bearing "ionic impurities" cause lattice defects^3^. This process explains why fossil samples from the Eocene have higher crystallite sizes (low FWHM values in Fig. 9a) but more lattice defects than Quaternary samples (blueshifts of v1-PO_4_^3−^ in Fig. 9a).

Our results corroborate the findings of Person et al.^74^ Thomas et al.^3^ and Piga et al.^46^. They show that the crystallite size and structural order do not have a strict relationship with the fossil age but with its diagenetic history and the geochemical microenvironment. The recrystallization process will occur in a microenvironment with other ionic constituents, which explains the relationship between the crystallinity and blueshift of v1-PO_4_^3−^ (Supplementary Fig. S6).

In the *LAPA/SBF-3-0017* sample, organic and mineral matrix decomposition is a source of the anion H_2_PO^4−^, and dripping water rich in calcium carbonate is a source of Ca^2+^. Collagen loss^71^ and dripping water that is rich in calcium carbonate will favour P, and Ca's demands for the new recrystallized mineral assume a structure similar to that of unaltered bone. This process occurs for the *LAPA/SBF-3-0017*. The data (Fig. 9b) indicate that the dissolution of biogenic phosphate and recrystallization of secondary hydroxyapatite occurred with a PO_4_^3−^/B-type CO_3_^2−^ ratio that is similar to that of the unaltered bone sample (Fig. 9b).

In a more advanced-stage calcium carbonate impregnation (Fig. 7d), the new calcite minerals will fill the cortical bone micropores and decrease the fossil permeability. This process was shown in a previous work^12^. From this point, water will accumulate on the fossil surface, which helps to form a calcium carbonate crust. The precipitation of carbonate minerals inside the bone structure and its external surface cause a basic microenvironment and physical protection from external agents. This protection will contribute to the maintenance of a minute organic fraction.

When the endmember of the diagenetic route has dripping water that is rich in calcium carbonate, significant biological structural changes occur in both the outer bone and the inner bone. The neoformation of hydroxyapatite crystals will occur by the dissolution and recrystallization process, as noted by Silen and Parkington^75^, Nielsen-Marsh and Hedges^1^, Karkanas et al.^76^, Berna et al.^2^, Trueman et al.^55^, due to the existence of enough sources of calcium and phosphorus in neutral or narrow alkaline pH conditions. The newly formed minerals will have a well-developed crystallite size with a high structural order. Its crystallographic structure resembles unaltered bones, with the B-type and A-type carbonate groups.

We employed the *LAPA/SBF-3-0081* sample as a model for the diagenetic processes observed in route B (Fig. 7e). This sample has a different taphonomic history than the *LAPA/SBF-3-0017* specimen, which is mainly related to the absence of dripping water that is rich in calcium carbonate and a weak influence of deposition events. Calcium carbonate precipitate is absent within the bone structure, as corroborated by Raman spectroscopy (Figs. 2 and 3 and Supplementary Fig. S2), SEM–EDS (Figs. 4, 5, and Supplementary Figs. S3, S4, and S5), and X-ray diffraction analyses (Fig. 6 and Supplementary Table S2). We observe only a few sediments with microgranular structures within the bone structure, which is composed of silt and clay, such as the clastic material detected in the *LAPA/SBF-3-0017* sample (Supplementary Fig. S1).

After sharing the same steps in the early stage of diagenesis (Fig. 7a,b) with *LAPA/SBF-3-0017*, in the *LAPA/SBF-3-0081* sample, a degradation process of the organic matrix occurred in the dry microenvironment (or even with the lowest humidity), which enabled the remarkable preservation of biological structures (Fig. 5). In the advanced stage of diagenesis, the decomposition of the (bio)mineral matrix led to the removal of the B-type carbonate from the hydroxyapatite (Figs. 7e and 9b), as described by Dal Sasso et al.^6^ and is observed by the increase in the relative intensity of v1-PO_4_^3−^ compared to the B-type CO_3_^2−^ (Fig. 9b). However, this observation suggests the formation of secondary hydroxyapatite.

The absence of dripping water that is rich in calcium carbonate implies an insufficient calcium source in the newly formed hydroxyapatite, which creates unfilled B-type carbonate sites. This process explains the largest ratio between PO_4_^3−^/B-type CO_3_^2−^ and the *LAPA/SBF-3-0017* sample (Fig. 9b). The blueshift of v1-PO_4_^3−^ observed in Fig. 9a indicates that the vacant B-type carbonate group sites were most likely occupied by other ionic constituents, which culminates in hydroxyapatite with minor crystallites and more structural disorder than the *LAPA/SBF-3-0017* sample. The diagenesis of hydroxyapatite in this route involves crystal growth but not necessarily dissolution of the original material, which enables better preservation of the biological structure (Fig. 5). Crystal growth may occur by nucleation^77^, which generates a nonbiogenic phosphate with a smaller crystallite size (Supplementary Table S2). Therefore, although the organic and inorganic phases decompose, the new mineral phase is precipitated in the space that is vacated by the previous mineral or in vascular structures.

As an endmember of diagenetic route B, another type of fossil is observed in tropical limestone caves. Fossils with preserved histological structures, cracks and broken bone features without secondary mineralization of calcite and total degradation of the organic fraction are commonly noticed^23^. However, the diagenetic process witnessed in crystallite size changes and structural disorder due to the removal of B-type carbonate groups was unknown. We emphasize that the hydroxyapatite crystallite sizes for the LAPA/SBF-3-0017 sample (ranges from 51.03 to 41.08 nm) and LAPA/SBF-3-0081 sample (ranges from 30.9 to 33.33 nm) obtained by Scherrer's equation (refer to Supplementary Table S2) are consistent with the data in the literature. Londoño-Restrepo et al.^47^ showed an average size of 25 nm for unaltered bovine, porcine, and human bone. For fresh bones, Dal Sasso et al.^71^ discovered sizes between 17.2 and 19.1 nm, and for archaeological bones, the values varied between 34 and 71 nm, which denotes the effect of diagenesis in increasing the crystallite size by the recrystallization process. However, Piga et al.^78^ show a minor effect of diagenesis on the increase in crystallite which varies from 16.9 to 28 nm for fossil bones from the Holocene to the Lower Pleistocene. Both our data and the data of Dal Sasso et al.^71^ and Piga et al.^78^ denote the influence of diagenesis conditions on crystallite size modification pathways.

Recrystallization of hydroxyapatite can alter the primary isotopic composition of fossil bone, which influences the original diet-derived isotopic signature^79, 80^. Recognizing the extent of diagenesis in fossil bone is essential for applying stable isotopic analysis to paleoecology studies^81, 82^. Tomaz et al.^73^ proposed Raman scattering proxy diagenetic changes in the primary oxygen isotopic composition in Oligocene, Eocene, and Cretaceous fossils. They suggested that the values of blueshift for ν1-PO_4_^3−^ higher than 964.7 cm^−1^ and FWHM less than 13 cm^−1^ are reasonable parameters for identifying the changes in the oxygen isotopic composition. However, Thomas et al.^73^ disregarded carbon isotopic and quaternary fossil samples. This question of changes in the original carbon and oxygen isotopic signature in Pleistocene fossils related to the diagenetic routes remains open and needs to be clarified.

## Conclusions

This study shows the diagenetic processes of Quaternary fossils that are commonly discovered in Brazilian limestone caves. We demonstrate the relationship between fossil diagenetic pathways and hydroxyapatite size and lattice changes.

We propose a model to explain the diagenetic process that includes an early stage and two routes of the advanced stage of diagenesis, which are referred to as routes A and B. The diagenetic route A endmember is characterized by calcite permineralization and hydroxyapatite recrystallization with high structural order and large crystallite size. Concerning the diagenetic endmember of route B, another type of fossil is observed. Fossils show well-preserved histology, with cracks and broken bone features and without calcium carbonate precipitation. The absence of carbonate-rich drip water implies a limited source of calcium during reprecipitation and nucleation of hydroxyapatite. In this case, the B-type carbonate group sites that are becoming unfilled lead to minor crystallite sizes and more significant structural disorder than the route A’s end-members.

Using raman spectroscopy, X-ray diffraction, and scanning electron microscopy, this study opens a new perspective for further research on the fossil diagenetic process in tropical environments, especially in limestone caves. New questions need to be answered to build a more robust diagenetic model: (1) What are the fossil diagenetic pathways in a route that comprise the oxyreduction cycle due to water flooding? (2) What will be the hydroxyapatite crystallinity changes in buried fossils in cave sediments? (3) Can diagenesis affect the carbon and oxygen isotope geochemistry in quaternary fossils? (4) Can the secondary calcite in the bone structure make some chemical bonds with hydroxyapatite and cause structural changes?

## Methods

We analyzed fossil samples and thin sections of the bones from *Valgipes bucklandi*, which is an extinct ground sloth, that were collected in different places of the ‘Gruta da Lapinha’ cave in the Nova Redenção municipality of Bahia state, Brazil. The fossiliferous material is deposited in the scientific collection of the Paleontology Laboratory (*LAPA/SBF*) of Federal University do São Francisco Valley (UNIVASF), Senhor do Bonfim campus in Bahia state, Brazil.

Two samples with different fossil diagenetic features that are commonly found in limestone Brazilian caves were chosen^40–44^. We classified the samples by their different taphonomic attributes (Supplementary Table S1) for each fossil-bearing site. The taxonomic diversity, which consists of taxa identification recorded in fossil accumulation, categorized the general bone representation, skeleton articulation, and taxa habitat. Weathering classification was determined from zero to five stages^83^, and abrasion was determined as no abrasion, moderate abrasion or heavy abrasion^84^.

Regarding breakage, for diverse types of fractures, we applied Shipman et al.^85^. A degree of breakage was added concerning the number of bone fractures, including low breakage (1 or 2 fractures per bone), medium breakage (2–5 fractures per bone), or high breakage (more than five fractures per bone), which follows Eltink et al.^46^. We analyzed three stages of incrustation, according to Maldonado et al.^85^, and we apply Voorhies’ groups^86^ for the aspects of transport we.

Different taphonomic features of each sample displayed distinctive characteristics in terms of diagenetic events. The *LAPA/SBF-3-0017* sample represents a well-altered fossil bone that demonstrates the strong influence of calcium carbonate secondary incrustation. On the other hand, the *LAPA/SBF-3-0081* sample stood for a weakly altered fossil bone. The next step was to perform lead spectrometry and geochemical analyses, which resulted in the relationship between these cave fossils.

### Sample preparation

In principle, microspectrometry techniques and X-ray diffraction are nondestructive methods, and sample preparation is not required. However, the assessment of diagenetic features inside the fossil bones required a thin section of cross-cutting rock lamination and polishing samples. Afterward, the samples passed the impregnation of epoxy resin and cutting by ultramicrotome. The sections were then polished using 1000, 2500, and 4000 grit sandpaper on a rotation stage with water as the lubricant, and subsequently, 1 µm diamond suspension on a polishing cloth. For X-ray diffraction analyses, we took a subsample of different regions of the samples *LAPA/SBF-3-0017* and *LAPA/SBF-3-0081* (Fig. 6). These subsamples were ground in a porcelain mortar until they reached a particle size between 100 and 200 mesh.

### Analytical techniques

The two thin sections of the *LAPA/SBF-3-0017* and *LAPA/SBF-3-0081* samples and two bone fragments of sample *LAPA/SBF-3-0017* were metalized with gold for analysis with a scanning electron microscope (SEM) (Vega 3XM Tescan using accelerations of 15 and 10 kV) that was equipped with an energy dispersive spectroscopy (EDS) detector, in which measurements were performed to obtain geochemical maps and point-source analyses.

Twenty-six raman spectroscopy measurements were carried out in different regions of the samples; these analyses were performed using a Renishaw micro-Raman inVia spectrometer that was equipped with a diode laser at 785 nm. This specific choice of the laser line is aimed at minor fluorescence effects. We obtained the spectra using a 50× objective and NA = 0.75. The micro-Raman measurements have a laser spot size of approximately 1 μm. The spectra baseline was extracted using a computational cubic mathematical model for all samples, and Lorentzian curve fitting was employed for the adjustment of all raman bands. After removal of the baseline, the remaining bands of interest may consist of one or several peaks. The frequency of the bands used in the deconvolution process to identify bone materials and calcite peaks was taken from the following reference samples: modern and unaltered pig bone, two spectra of apatite from the American Mineralogist Crystal Structure Database (http://rruff.geo.arizona.edu/AMS/amcsd.php), R050512 and R060180, and seven spectra of hydroxyapatite. Details are provided in Supplementary Table S5.

To evaluate the effect of diagenesis on the bone preservation structure, we calculated the following parameters from Raman spectra: (1) full width at half maximum (FWHM), (2) peak position, and (3) ratio of v1-PO_4_^3−^/v1-CO_3_^2−^ B-type. We use these parameters as proxies of diagenetic alteration. We compare our FWHM data and peak positions with the literature on natural amorphous calcium phosphate, synthetic hydroxyapatite, unchanged bone, geologic apatite, Holocene, Oligocene, Mid Eocene, and Late Cretaceous fossils (Supplementary Table S5).

X-ray diffraction (XRD) measurements were taken with a powder diffractometer Bruker X-ray Diffractometer, model D8 Advance Davinci, with Cu-Kα radiation (λ = 1.5418 Å), with the tube operating at 40 kV/40 mA in continuous mode at room temperature. The crystalline phases were identified using the software X-Pert HighScore (Panalytical)^87^, We obtained crystallography data for all phases using the inorganic crystal structure database (ICSD). We calculated the lattice parameter of crystallite size by the Scherrer equation^88^.

## Supplementary information

Supplementary information.
