# Supplementary material for: Diagenetic processes in Quaternary fossil bones from tropical limestone caves
Source: Sci Rep. 2020 Dec 8;10:21425. doi: 10.1038/s41598-020-78482-0 (PMC7722736; doi:10.1038/s41598-020-78482-0)
Supplement: Supplementary file 1 — Supplementary information. [file 41598_2020_78482_MOESM1_ESM.pdf]

Diagenetic processes in Quaternary fossil bones from tropical limestone caves

Daniel Vieira de Sousa[1]*, Estevan Eltink[2], Raquel Aline Pessoa Oliveira[3], Jorlandio Francisco Felix[4], Luciano Moura Guimarães[5]

[1] Colegiado de Geografia, Universidade Federal do Vale do São Francisco, Senhor do Bonfim, 48970-000, Brasil
[2] Colegiado de Ecologia, Universidade Federal do Vale do São Francisco, Senhor do Bonfim, 48970-000, Brasil
[3] Colegiado de Ciências dos Materiais, Universidade Federal do Vale do São Francisco, Juazeiro, 48902-300
[4] Instituto de Física, Universidade de Brasília, Brasília, 70910-900, Brasil
[5] Departamento de Física, Universidade Federal de Viçosa, Viçosa, 36570-000, Brasil

*daniel.vsousa@univasf.edu.br

Supplementary Material

Supplementary Table S1. Taphonomic features of the samples

	Sample 1 - LAPA/SBF-3-0017 - site 17 (Lateral hall of colluvium ramp)	Sample 2 - LAPA/SBF-3-0081 - Site 8 (Benevides Hall)
Taxonomic representation	Scelidotheriinae<br><br>*Valgipes bucklandi*	Scelidotheriinae<br><br>*Valgipes bucklandi*
Bone representation	Vertebra	Vertebra*
Habitats	Terrestrial	Terrestrial
Articulation of skeletal elements	Isolated	Disarticulated
Weathering	Stage 1	Stage 0 and 1
Lichen impression	No	No
Abrasion	No abrasion/moderate	No abrasion/moderate
Breakage types	E and F	E, F and G
Breakage degree	Medium	Medium
Incrustation	Stage 2	Stage 0
Transport	Voorhies I	Voorhies I, II and III
Staining	Darkish and reddish	Whitish and reddish

(*) From almost complete skeleton with mandible, fragmentary long bones of hind and forelimbs elements, ribs, astragalus and calcaneum.

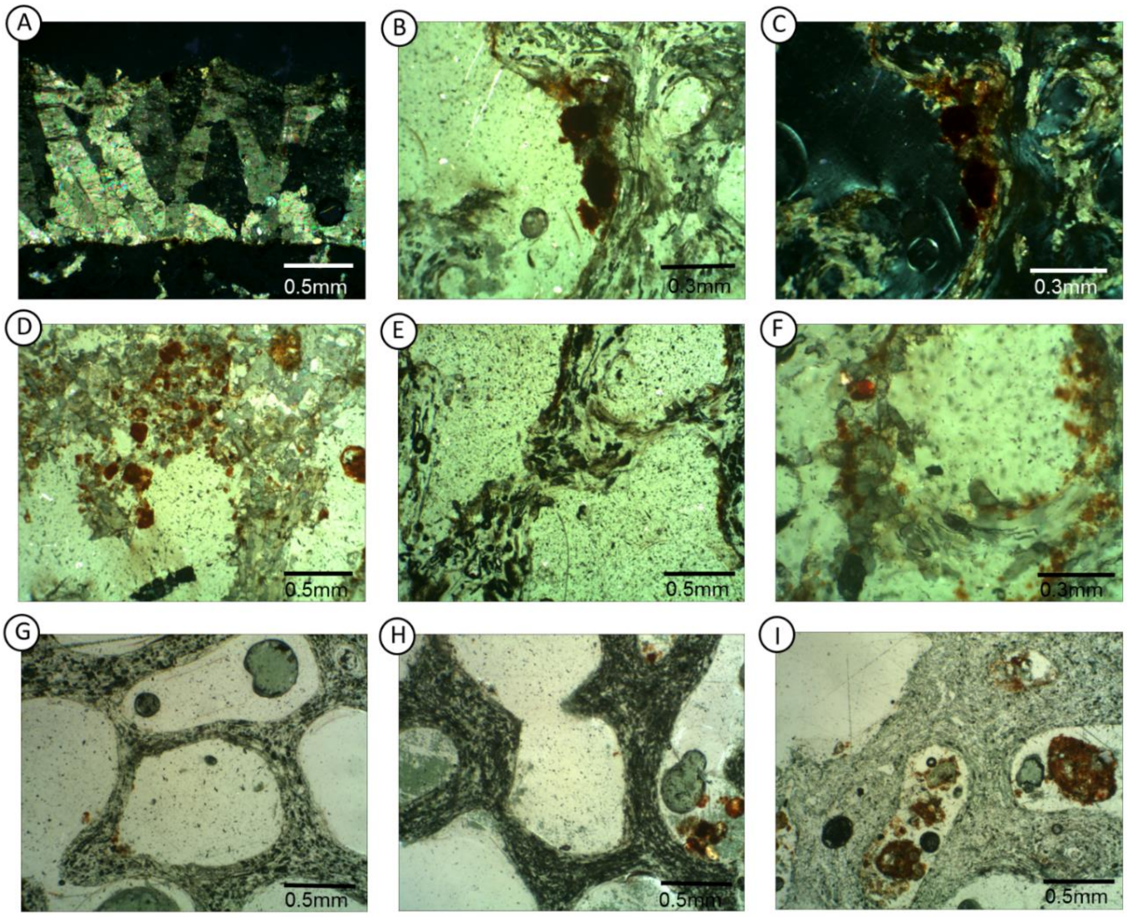

Supplementary Fig. S1. Micromorphological features of the samples *LAPA/SBF-3-0017* and *LAPA/SBF-3-0081* observed under polarized microscopy. A – Calcite precipitation in outer surface of sample *LAPA/SBF-3-0017*, image in XPL. B and C – Details of bone features of the *LAPA/SBF-3-0017*, as some microgranular like soil structures composed of iron oxide (B), and precipitation of calcium carbonate in the bone pores (C), image in XPL. D, E, and F – Cancellous bone of the sample *LAPA/SBF-3-0017*, with microgranular like structure composed by iron oxide (D, F). G, H, and I – Cancellous bone structures of the sample *LAPA/SBF-3-0081*, with microgranular like soil structures (H, I).

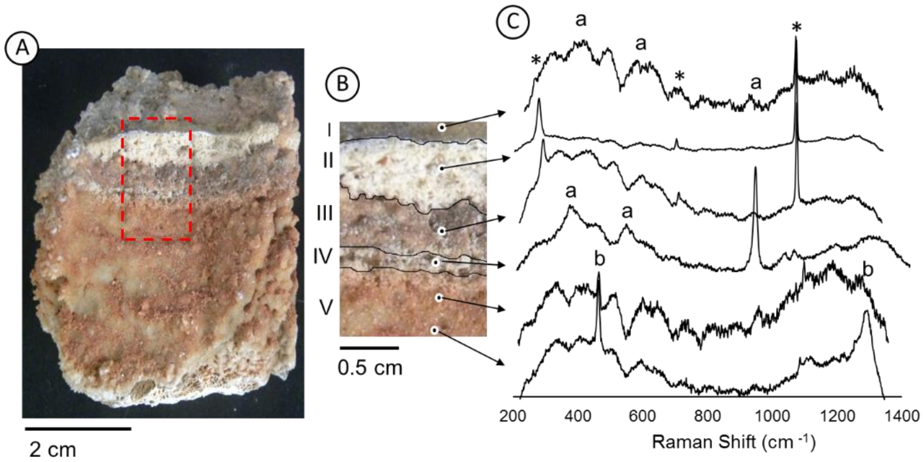

Supplementary Fig. S2. Detailed microstratigraphy present on the external surface of *LAPA/SBF-3-0017* sample (A and B) and band assignments of micro-Raman spectroscopy corresponding to each layer (C). A - Region of the vertebra (red box) where five layers (I, II, III, IV, V) selected for micro-Raman Spectroscopy analysis. B -Detailing of layers and the aimed points for micro-Raman spectroscopy (black dots). C - Spectra of each microstratigraphic layer. The peaks marked with (*) show the presence of calcite; the peaks marked (a) indicate the presence of phosphate, the peaks indicated with (b) indicate the presence of hematite.

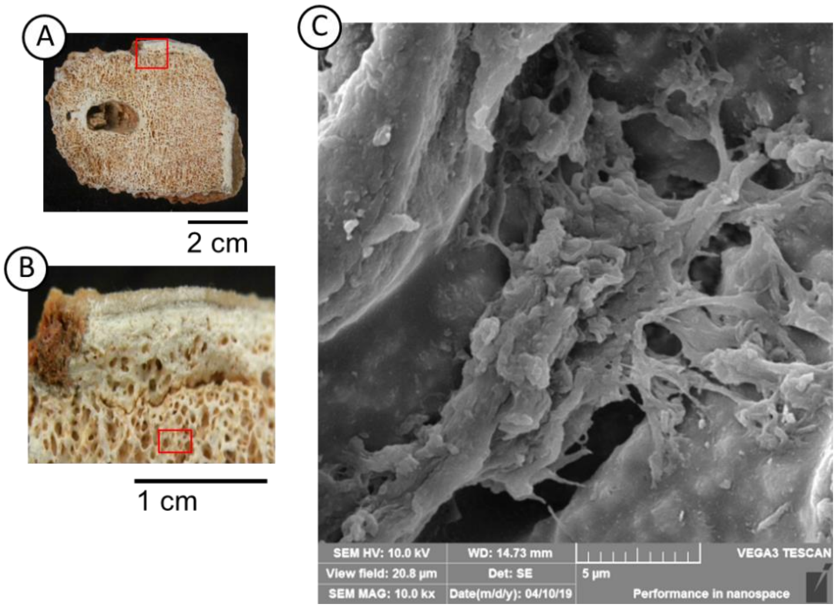

Supplementary Fig. S3. Scanning electron microscopy of the sample *LAPA/SBF-3-0017* . A - View of the sample, the red box indicates the location of magnification of image B; B - Detailed view of the different diagenetic facies, the red box indicates the location of the measurement in electronic spectroscopy, which can be seen in image C. C - Collagen-like structures.

Supplementary Table S2. Data of samples *LAPA/SBF-3-0017* and *LAPA/SBF-3-0081* used in Scherrer equation to calculate the crystallite size

	------------------------------ **LAPA/SBF-3-0017** ------------------------------								---------------------- **LAPA/SBF-3-0081** ----------------------					
	1 - external surface		2 - external surface		3 - internal surface		4 - internal surface		5- external surface		6 - external surface		7 - external surface	
	2 θ°	Cristalite size (nm)	2 θ°	Cristalite size (nm)	2 θ°	Cristalite size (nm)	2 θ°	Cristalite size (nm)	2 θ°	Cristalite size (nm)	2 θ°	Cristalite size (nm)	2 θ°	Cristalite size (nm)
	23.14	66.42	12.33	23.26	23.08	31.80	10.82	18.46	10.86	31.30	10.76	23.22	10.84	23.23
	26.68	66.88	20.88	66.17	25.87	66.76	23.17	66.4	25.87	48.99	22.88	15.56	25.85	48.98
	29.50	49.37	23.14	66.42	29.47	49.37	25.90	49.00	26.65	49.06	25.86	48.99	28.16	32.12
	31.58	67.62	25.90	48.99	31.77	27.60	28.90	32.17	28.11	67.0	28.09	49.22	28.93	32.18
	36.09	68.44	26.70	66.88	32.20	67.73	29.51	49.37	29.04	32.18	29.03	23.88	31.81	32.40
	39.53	69.15	27.50	66.70	32.85	32.48	31.80	39.21	31.80	39.21	31.76	39.20	32.21	49.70
	43.29	51.37	29.52	67.29	34.04	24.18	32.97	24.11	32.21	49.70	32.22	67.73	32.94	32.49
	47.26	52.11	31.74	27.60	36.05	39.65	34.09	32.59	33.07	24.12	32.95	32.49	34.08	24.18
	47.68	52.20	32.95	19.17	39.49	69.14	36.07	32.77	34.14	32.59	34.09	39.44	39.91	24.60
	48.66	52.40	36.08	68.44	43.26	51.36	39.90	16.23	39.95	24.60	39.89	24.60	42.22	12.20
	56.72	35.41	39.54	69.15	46.71	33.94	42.33	10.83	42.12	16.35	42.13	16.35	46.70	41.07
	57.56	43.02	43.30	51.37	49.48	25.46	43.29	70.01	43.86	33.59	43.90	16.45	48.09	25.32
	60.81	55.36	47.69	52.20	53.20	53.40	46.73	52.01	46.83	33.95	46.71	33.94	49.48	29.23
	61.56	55.57	48.66	52.40	61.57	13.25	48.68	71.42	48.20	25.33	48.19	25.33	50.59	34.46
	63.14	17.90	49.53	34.31	64.90	30.05	49.54	29.23	49.50	41.52	49.49	34.31	53.11	42.16
	64.81	56.55	50.20	52.73			53.23	34.27	50.65	34.47	50.61	25.58	55.98	14.11
	65.84	56.87	53.19	34.84					53.13	42.16	52.20	25.75		
	73.14	28.80	57.55	74.24					55.98	17.28	53.10	42.15		

	77.39	24.16	60.04	35.98					60.30	13.16	60.28	13.16		
			60.90	36.14					64.16	27.29	61.66	36.28		
									77.12	15.94	77.12	15.94		
**MCS (nm)** [1]	**51.03**		**50.73**		**41.08**		**39.26**		**33.33**		**30.93**		**31.15**	

1 – Medium crystallite size

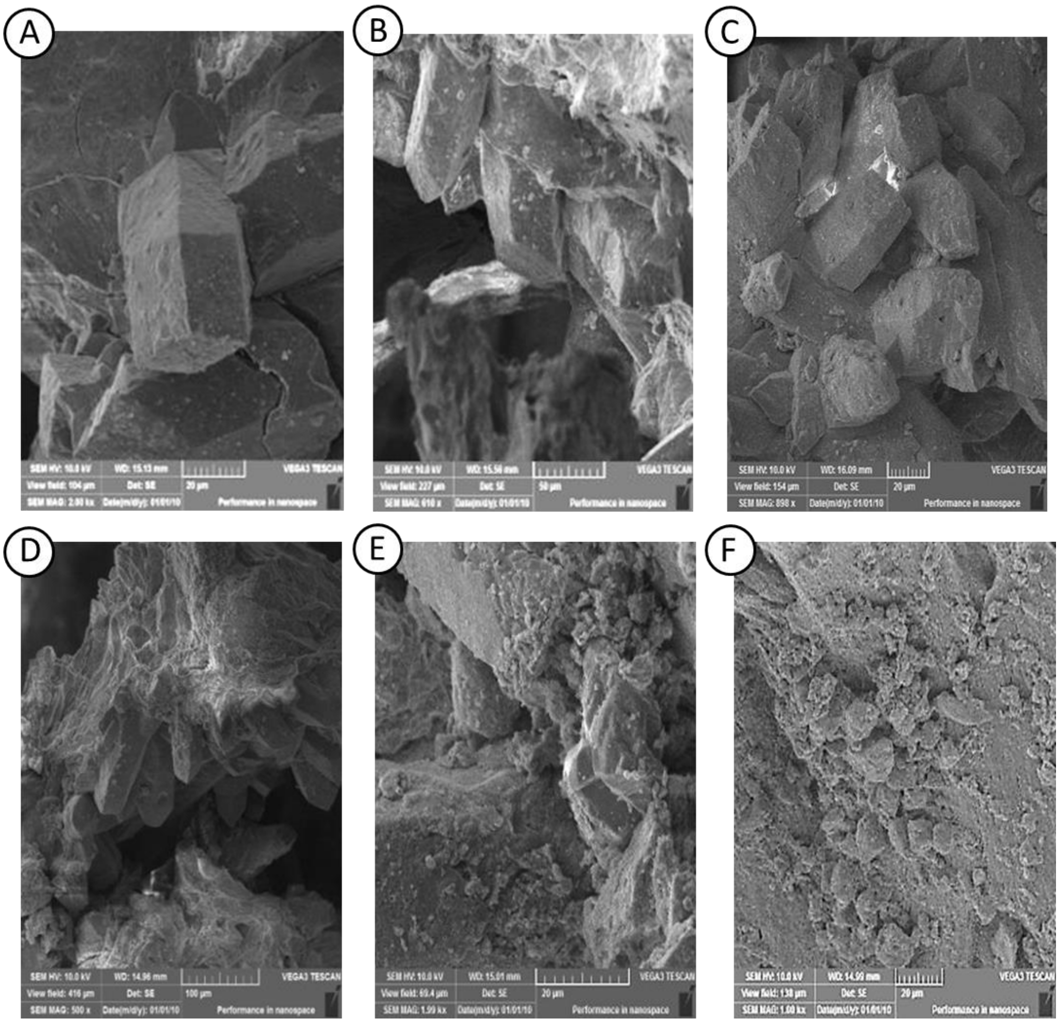

Supplementary Fig. S4. Neoformation of calcite inside of porous space of the sample *LAPA/SBF-3-0017.* A, B, C, D - Well-developed crystals of calcite. E, F - Terrigenous sediment composed by iron oxides on this crystallized calcite in the porous space.

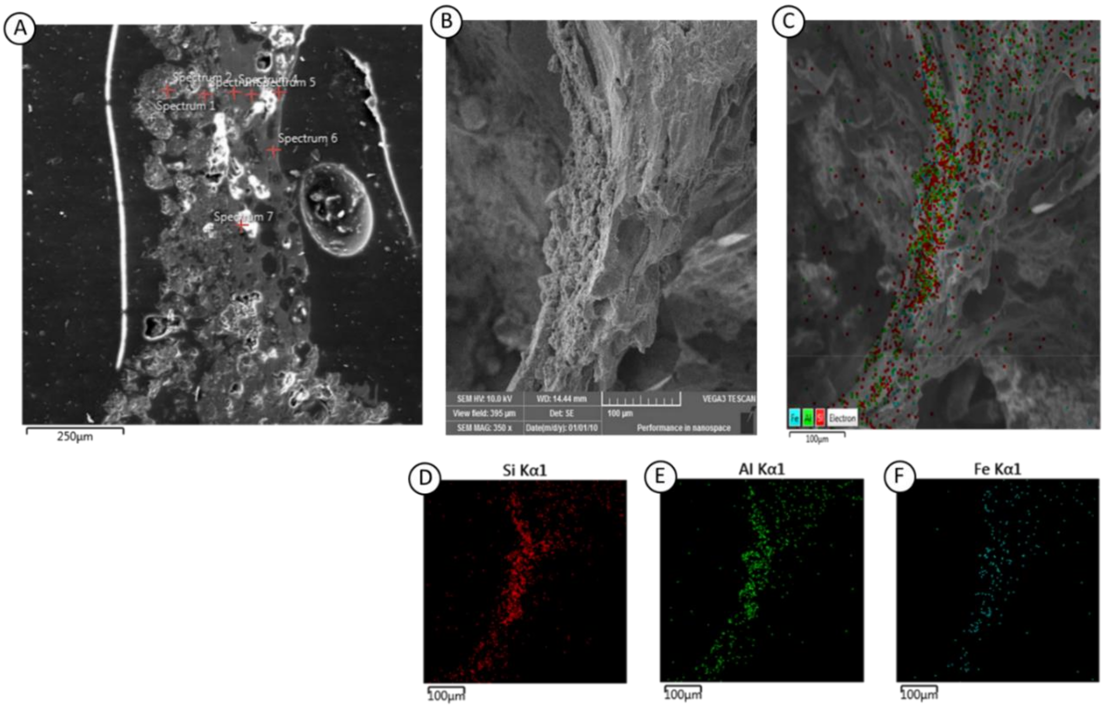

Supplementary Fig. S5. - Scanning Electron Microscopy images of the sample *LAPA/SBF-3-0017* suggest the erosion of well development soil mantles like Oxisols and Acrisols, with predominance of kaolinitic and oxidic mineralogy. A – Backscatering image and microchemical point analyses showed in Supplemental Table 3. B – Electron secondary image. C, D, E, F- Microchemical mapping of coating clay material revealsthe clay composition encompassed of silica, aluminum and iron oxide, suggesting the kaolinite and oxide composition.

Supplementary Table S3. Microchemical point analysis of Supplemental Figure 5 A.

Imagem	Point	Element %											
		O	Ca	P	Al	Fe	Na	Si	Cl	Nb	K	Mg	Ti
	1	54.7	0.1					44.9	0.1		0.1		
	2	49.3						50.7					
	3	44	1		18.4	5.8		22.3	0.2		0.7	0.7	0.7
Supplementary Fig. S5 A.	4	44.6	37.1	17.9			0.4						
	5	45.3	36.6	17.7			0.5						
	6	43.9	37.6	18			0.5						
	7	24.7	1		18.5	9.3	1.4	37.9			3.3		12.7

Supplementary Table S4. Our data and data gathered from literature (Fig 9 A) of Raman spectrum of fossil bone. The FWHM and center are the v1-PO_4^{-3} vibrational mode.

F WHM	Center	Sample	Source
31	951	Natural ACP	Grauw 1996 [ref. S1]
26,87	950,9	Syntetic Amorphous Calcium Phosphate	Stammeier et al 2018 [ref. S2]
23,14	950	Syntetic Amorphous Calcium Phosphate	Stammeier et al 2018
13,66	959,8	Syntetic hidroxyapatite Sigma Aldrich	Stammeier et al 2018
16,76	959,3	Syntitic Hydroxyapatite	Stammeier et al 2018
16,6632	959	Natural bone	Dooley 2011 [ref. S3]
18,51201	958,3	Natural bone	Raghavan 2011 [ref. S4]
18,5555	958	Humam bone	Khalid et al 2018 [ref. S5]
14,4764	958,59	Pig Bone	This work
8,7	965	Late Cretaceus Dentin	Thomas et al 2011 [ref. S6]
11,8	966,5	Striatolamia sp. Mid eocene, dentin)	Thomas et al 2011
12	965	Oligocene Fossil	Thomas et al., 2007 [ref. S7]
16	963	Holocene sub-fossil	Thomas et al., 2007
3,5	965,4	Magmatic fluorapatite	Thomas et al 2011
4,3	965,5	Magmatic fluorapatite	Thomas et al 2011
8,5	962,7	Magmatic hydroxylapatite	Thomas et al 2011
7,5468	962,6931	RRUFF ID R130713	Robert Lavisnk [ref. S8]
7,0933	961,3117	RRUFF ID R100225	Luiz Menezes [ref. S8]
6,452	962,6094	RRUFF ID RO 60180	Julian Gray [ref. S8]
7,9029	961,4244	RRUFF ID R050512	American Museum of Natural History 15214 [ref. S8]
13,4573	965,0421	LAPA/SBF-3-0081 external surface	This work
12,4646	962,4927	LAPA/SBF-3-0081 external surface	This work
11,6446	961,7878	LAPA/SBF-3-0081 external surface	This work
11,8671	961,1146	LAPA/SBF-3-0081 external surface	This work
9,6504	961,0684	LAPA/SBF-3-0081 internal surface, cortical bone	This work
9,6453	961,1517	LAPA/SBF-3-0081 internal surface, cortical bone	This work
9,8399	961,1348	LAPA/SBF-3-0081 internal surface, cortical bone	This work
13,3425	960,6548	LAPA/SBF-3-0081 internal surface, cancelous bone	This work
11,2591	960,9948	LAPA/SBF-3-0081 internal surface, cancelous bone	This work
11,3424	960,686	LAPA/SBF-3-0081 internal surface, cancelous bone	This work
10,3425	959,5133	LAPA/SBF-3-0017, interface betewen edge and internal surface	This work
9,8046	960,7624	LAPA/SBF-3-0017, internal surface, cortical bone	This work
9,948	960,5635	LAPA/SBF-3-0017, internal surface, cortical bone	This work
9,7923	960,0599	LAPA/SBF-3-0017, internal surface, cortical bone	This work
9,9206	959,7064	LAPA/SBF-3-0017, internal surface, cancelous bone	This work
9,3468	959,9862	LAPA/SBF-3-0017, internal surface, cancelous bone	This work
9,5918	959,908	LAPA/SBF-3-0017, external surface, cancelous bone	This work
9,781	959,976	LAPA/SBF-3-0017, external surface, cancelous bone	This work
9,8873	959,9722	LAPA/SBF-3-0017, external surface, cancelous bone	This work
16,0534	959,3387	LAPA/SBF-3-0017, internal surface, cortical bone	This work
12,4915	960,2033	LAPA/SBF-3-0017, internal surface, cortical bone	This work

16,4022	959,8969	LAPA/SBF-3-0017, external surface, cancelous bone	This work
10,1711	959,2952	LAPA/SBF-3-0017, internal surface, cortical bone	This work
10,1125	959,2773	LAPA/SBF-3-0017, internal surface, cortical bone	This work
11,2603	959,2207	LAPA/SBF-3-0017, external surface, cancelous bone	This work
10,5625	959,951	LAPA/SBF-3-0017, external surface, cancelous bone	This work

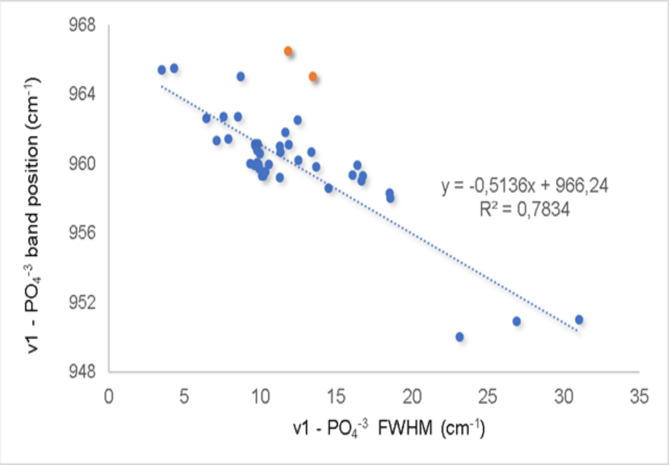

Supplementary Fig. S6. Relationship between the crystallinity and blueshift of the v1-PO_4^{-3}. This chart was construct with the data shown in the Supplemental Table 4. The orange points were considered as outliers and not considered in the regression analysis.

Supplemental Table S5. Reference of Apatite.

Sample	Reference
Natural and Synthetic Amorphous Calcium Phosphate	Grauw 1996; Stammeier et al. (2018) [ref. S1, S2]
Syntetic Hydroxyapatite	Stammeier et al 2018 [ref. S2]
Natural bone	Dooley (2011); Mekhala Raghavan (2011); Khalid et al. (2018) [ref. S3, S4, S5]
Holocene sub-fossil	Thomas et al., 2007 [ref. S7]
Oligocene Fossil	Thomas et al., 2007
Mid Eocene Fossil	Thomas et al., 2011 [ref. S6]
Late Cretaceous Fossil	Thomas et al., 2011
RRUFF - Geologic Hydroxylapatite	Thomas et al., 2011; Robert Lavinsk; Luiz Menezes; Julian Gray; American Museum of Natural History 15214 [ref. S6, S8]

Supplementary References S1-S8

S1. De Grauw, C. J., De Bruijn, J. D., Otto, C., Greve, J. & De Grauw, K. Investigation of bone and calcium phosphate coatings and crystallinity determination using raman microspectroscopy. *Cells Mater.* (1996).

S2. Stammeier, J. A., Purgstaller, B., Hippler, D., Mavromatis, V. & Dietzel, M. In-situ Raman spectroscopy of amorphous calcium phosphate to crystalline hydroxyapatite transformation. *MethodsX* (2018) doi:10.1016/j.mex.2018.09.015.

S3. Dooley, Kathryn A. Raman Spectroscopic Studies of Bone Biomechanical Function and Development in Animal Models. 2011 PhD Thesis.

S4. Mekhala Raghavan. Investigation of mineral and collagen organization in bone using raman spectroscopy 2011. PhD. Thesis

S.5 Khalid, M., Bora, T., Ghaithi, A. Al, Thukral, S. & Dutta, J. Raman spectroscopy detects changes in bone mineral quality and collagen cross-linkage in staphylococcus infected human bone. *Sci. Rep.* (2018) doi:10.1038/s41598-018-27752-z.

S6. Thomas, D. B., Fordyce, R. E., Frew, R. D. & Gordon, K. C. A rapid, non-destructive method of detecting diagenetic alteration in fossil bone using Raman spectroscopy. *J. Raman Spectrosc.* (2007). doi:10.1002/jrs.1851

S7. Thomas, D. B., McGoverin, C. M., Fordyce, R. E., Frew, R. D. & Gordon, K. C. Raman spectroscopy of fossil bioapatite - A proxy for diagenetic alteration of the oxygen isotope composition. *Palaeogeogr. Palaeoclimatol. Palaeoecol.* (2011) doi:10.1016/j.palaeo.2011.06.016.

S8. Lafuente B, Downs R T, Yang H, Stone N (2015) The power of databases: the RRUFF project. In: Highlights in Mineralogical Crystallography, T Armbruster and R M Danisi, eds. Berlin, Germany, W. De Gruyter, pp 1-30
